# Ping pong fracture

**DOI:** 10.1093/omcr/omae093

**Published:** 2024-08-23

**Authors:** Boumeriem Khaoula, Bourekba Iliass, Ait Belhaj El Mahdi, Allali Nazik, Chat Latifa, El Haddad Siham

**Affiliations:** Department of Pediatric Radiology, Pediatric Hospital, Mfadel Cherkaoui Street, Souissi, Rabat, Morocco; Department of Pediatric Radiology, Pediatric Hospital, Mfadel Cherkaoui Street, Souissi, Rabat, Morocco; Department of Pediatric Radiology, Pediatric Hospital, Mfadel Cherkaoui Street, Souissi, Rabat, Morocco; Department of Pediatric Radiology, Pediatric Hospital, Mfadel Cherkaoui Street, Souissi, Rabat, Morocco; Department of Pediatric Radiology, Pediatric Hospital, Mfadel Cherkaoui Street, Souissi, Rabat, Morocco; Department of Pediatric Radiology, Pediatric Hospital, Mfadel Cherkaoui Street, Souissi, Rabat, Morocco

**Figure 1:**
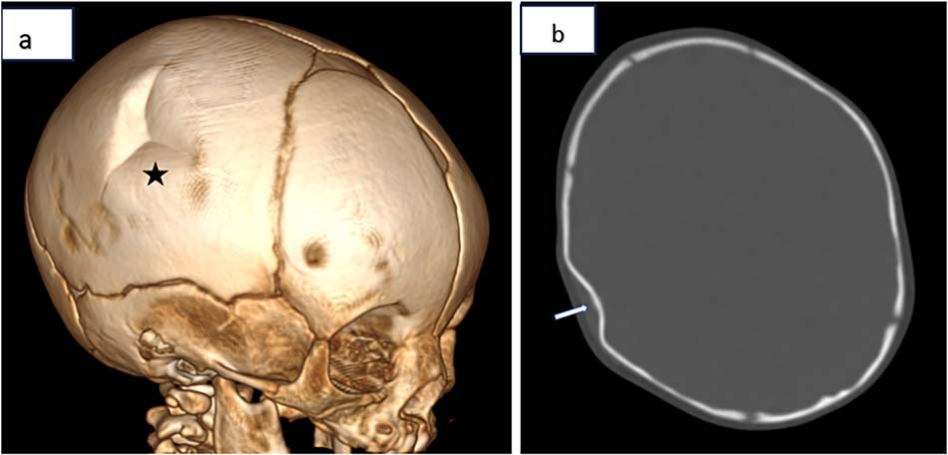
(**a** and **b**) Three-dimensional volume-rendered reconstruction and Axial CT showing the typical appearance of a ping-pong ball fracture (Star) and a depression of the right parietal bone consistent with a ping-pong ball fracture (Arrow).

A two-months infant, with no particular medical history, admitted to the emergency department for suspicion of cranial trauma, the physical examination revealed a deformation of the right parietal bone, he then underwent a brain CT scan that showed a depression in the right parietal bone consistent with a typical ping-pong fracture (Figure 1).

A ping-pong ball fracture is an inward depression of the calvarium occurring in newborns and infants due to the malleability of the skull at this age, generally resulting from cranial trauma or traumatic events occurring during the in utero phase or labor [1].

There are two main forms: congenital and acquired. Congenital fractures can occur either before birth or during delivery. Acquired fractures are related to obstetric interventions or postnatal trauma [1].

The diagnosis of a ping-pong ball fracture in a newborn is generally evident during physical examination, presenting as a unilateral depression, most often at the parietal vault, occasionally at the frontal region, and more rarely at the occipital region [2].

Radiological examinations confirm this diagnosis. A simple radiograph can show a focal deformation of the skull with inward indentation. Computed tomography (CT) is the reference examination to assess the extent and shape of the fracture and to exclude associated injuries; it reveals a cranial indentation without a clearly visible fracture line. CT is more sensitive than conventional radiography, reducing errors in interpreting sutures and vascular impressions. Transfontanellar ultrasound is sometimes useful for detecting parenchymal injuries or post-traumatic collections, while MRI is generally limited to cases with uncertain intracranial findings [3].

The therapeutic approach depend on the severity of the depression and the presence of any associated intracranial injury. Small fractures can resolve spontaneously or be treated with non-invasive techniques such as digital manipulation and vacuum devices. However, significant fractures generally require intervention [4].
